# Metabolic profile and behavior of clethodim and spirotetramat in herbs during plant growth and processing under controlled conditions

**DOI:** 10.1038/s41598-020-58130-3

**Published:** 2020-01-28

**Authors:** Magdalena Jankowska, Piotr Kaczyński, Bożena Łozowicka

**Affiliations:** Institute of Plant Protection - National Research Institute, Laboratory of Food and Feed Safety, Chełmońskiego 22, Postal code: 15-195 Bialystok, Poland

**Keywords:** Environmental monitoring, Process chemistry

## Abstract

Herbs may contain pesticide residues which are an important discriminator of food security and food quality. The challenge of the research was to assess the fate of the herbicide clethodim (CLE) and the insecticide spirotetramat (SPI) applied in herbs (BBCH 11-21) during herb growth and processing under controlled greenhouse trial conditions. The metabolic profile of CLE and SPI and their degradation products in basil (*Ocimum basilicum* L.), peppermint (*Mentha* × *piperita* L.) and sage (*Salvia officinalis* L.) was also presented. The half-lives of CLE and SPI in herbs were 1.10–1.56 days and 0.51–0.83 days, respectively. The terminal residues of SPI (SPI-enol, SPI-ketohydroxy, SPI-monohydroxy and SPI-enol-glucoside) and CLE (CLE-sulfone and CLE-sulfoxide) in herbal matrices were measured below EU maximum residue limits. In this paper, we aimed to assess the impact of washing and dehydratation pretreatment and calculated processing factors (PFs) which can be applied to more accurate food safety assessments. The PF values of CLE and SPI after drying prior washing was below 1 indicating reduction of initial residues. Drying process without washing demonstrated increases of SPI concentrations (PF up to 1.50). The lowest PFs were obtained when raw herbal plants were washed before drying showing almost complete degradation of parent compound (93–99%).

## Introduction

Herbal plants are widely used fresh materials for the food and health industries, especially dried herbs and spices are popular for consumers because they have multiple phytochemical properties^1^. These include phenolic compounds, alkaloids, flavonoids, anthocyanidins, cardenolides or tannins^2^. In addition to these essential and valuable substances, herbs contain contaminants, especially pesticide residues which are a critical discriminator of food quality and safety^3^. Pesticides belong to substances toxic for humans and some of them are mutagenic, teratogenic, carcinogenic and allergenic and persistent in the environment (they are not easily decomposed, they have the possibility of bioaccumulation and migration)^4^.

The maximum acceptable levels have been unified by various international organizations to assure the safety of raw herb commodities used as pharmaceutical products or food^5–8^. According to a report from the European Food Safety Authority^9^, 65.1% of the analyzed herbs contained mainly organophosphate and pyrethroid insecticides, 9.4% exceeded the Maximum Residue Limits set by the EU (MRLs = 0.01; 0.05 mg/kg). In herbal samples, insecticides (organophosphorus, chloroorganic and pyrethroid), fungicides and herbicides are detected at average concentration levels from 0.01 mg/kg up to 2.0 mg/kg^10–15^.

Clethodim is cyclohexanedione herbicide very often used for the selective control of the monocotyledonous weeds such as millet, thick oats in herbal plants. Insecticide, spirotetramat belongs to tetramic acid family and is very commonly used against aphids because herbal plants are very often attracted by this insect (Table S1). After these pesticides application, metabolic pathways and degradation processes may modify the toxicological properties of the active substance^16^. In some instances metabolites are more toxic and consequently pose a greater risk to the environment than the parent compound. Clethodim and spirotetramat both have a complex residue definition (CLE sum of clethodim, CLE-sulphoxide and CLE-sulphone and their 5-hydroxy conjugates; SPI sum of spirotetramat, (BYI08330) SPI-enol, SPI-ketohydroxy, SPI-monohydroxy and SPI-enol-glucoside). Only limited information on the toxicological properties of CLE/SPI-metabolites is available and are presented in citrus, *Appiacea* and *Brassicaceae* vegetables, colza and soil^17,18^. Thus, it is of great significance to evaluate the metabolic profile of CLE and SPI and their behavior during plant growth and processing. The combined exposure of CLE/ SPI and contribution of individual metabolites is needed in the toxicological characterization to make more accurate food safety assessments.

Herbs are commonly used medicinal means as a complementary or an alternative form of healthcare and are considered to be safe^19^. The World Health Organization (WHO) survey presented that most populations rely on non-conventional herbal medicines. Despite the presence of many health benefits of herbs, many contaminates in fresh commodities during cultivation, harvesting, processing and storage can occur and make their consumption unsafe^19^.

Technological processing is the method that changes the concentration of toxic substances in the processed product^20–22^. It might completely reduce the concentration, have no lack of influence and sometimes increase the levels in the final product^23–25^. Herbal raw materials, due to their short durability and seasonality, undergo various thermal treatments. Drying is the oldest method of preservation of food and the most frequently used way of preserving herbs that is the basis for their further use. It allows extending the durability of herbal products, preserving their taste, aroma, as well as many pro-health properties^26,27^.

The improvement of living standards showed that people increasingly care more about their health and pay attention to food safety. Herbs, especially in the dried form of e.g. spices, teas, pharmaceutical and cosmetics product are very popular for consumers^28^. Drying significantly reduces pesticide residues, regardless of whether the material is exposed to solar radiation or using drying equipment^29^. Thus, development of processing technologies for reduction of toxic substances in herbal commodities was a priority for this research.

To our best knowledge, the effect of food processing on CLE/SPI in herbs was not studied. The scientific literature is focused on individual groups of compounds (mainly organophosphorus) during the preparation of herbal infusions or cooking herbs^29–31^. The above facts clearly indicate that nowadays, it is an extremely important goal to evaluate the behavior of clethodim and spirotetramat.

Therefore, the science aim of the study was to: (1) evaluate metabolic profile of the cyclohexanedione herbicide and the tetramic acid insecticide in herbs during plant growth; (2) present dissipation dynamics of CLE and SPI in fresh basil, peppermint and sage (*Ocimum basilicum* L., *Mentha* × *piperita* L. and *Salvia officinalis* L.) and determine the half-lives; (3) investigate the residual of these pesticides and metabolites during processing of fresh herbal plants; (4) calculate the PFs of sum CLE and sum SPI in washing/drying process regarding to EU residue definition and (5) an additional scientific aspect of the work will be supplementation PFs databases for the first time for herbs.

## Results and Discussion

### Method performance

A modified QuEChERS method for simultaneous determination of SPI and CLE and their six metabolites in various species of herbal plants was applied followed by LC-MS/MS. The average recoveries of spirotetramat/SPI-metabolites in fresh and dried matrices were within 85–101% and 87–96%, respectively while for clethodim/CLE-metabolites in the range of 80–108% and 74–102%, respectively. These values were within the range expected for residue analysis. The reproducibility of recovery results, as indicated by relative standard deviations (RSDs) < 20%, confirmed that the method is sufficiently reliable for pesticide analysis in this study^32^. The calibration curve was linear over the concentration range with determination coefficients R^2^ > 0.995. The limit of detection (LOD) was 0.005 mg/kg whereas limit of quantification (LOQ) was 0.001 mg/kg for spirotetramat and clethodim and their metabolites.

### Metabolic profile – fate of clethodim

The initial residues of herbicide clethodim after 1 h of application in basil, peppermint and sage were 3.897 mg/kg, 4.251 mg/kg and 4.197 mg/kg, respectively (Table 1).

**Table 1:** Fate of clethodim in three species of herbs.

Days	Parent substance	Metabolites	
	clethodim	sulfone	sulfoxide
	Mean C ± SD n = 3	Mean C ± SD n = 3	Mean C ± SD n = 3
BASIL *Ocimum basilicum* L.			
0 (1 h)	3.897 ± 0.0034	0.246 ± 0.0056	1.965 ± 0.0046
1	0.728 ± 0.0028	0.504 ± 0.0061	1.257 ± 0.0035
2	0.459 ± 0.0046	0.493 ± 0.0021	0.787 ± 0.0043
3	0.293 ± 0.0054	0.305 ± 0.0029	0.505 ± 0.0028
7	0.270 ± 0.0019	0.239 ± 0.0017	0.299 ± 0.0019
14	0.251 ± 0.0022	0.105 ± 0.0026	0.220 ± 0.0015
21	0.151 ± 0.0030	0.046 ± 0.0010	0.139 ± 0.0011
PEPPERMINT *Mentha* × *piperita* L.			
0 (1 h)	4.251 ± 0.0042	0.220 ± 0.0036	1.948 ± 0.0068
1	0.203 ± 0.0028	0.316 ± 0.0025	0.602 ± 0.0044
2	0.198 ± 0.0033	0.492 ± 0.0029	0.310 ± 0.0026
3	0.175 ± 0.0019	0.318 ± 0.0031	0.373 ± 0.0031
7	0.084 ± 0.0023	0.150 ± 0.0016	0.136 ± 0.0027
14	0.060 ± 0.0017	0.079 ± 0.0013	0.078 ± 0.0014
21	<LOQ	0.033 ± 0.0009	0.022 ± 0.0008
SAGE *Salvia officinalis* L.			
0 (1 h)	4.197 ± 0.0048	0.221 ± 0.0060	2.437 ± 0.0055
1	0.983 ± 0.0052	0.253 ± 0.0042	0.304 ± 0.0021
2	0.917 ± 0.0035	0.431 ± 0.0021	0.112 ± 0.0032
3	0.444 ± 0.0031	0.328 ± 0.0044	0.064 ± 0.0011
7	0.447 ± 0.0014	0.302 ± 0.0020	0.026 ± 0.0018
14	<LOQ	0.204 ± 0.0031	0.009 ± 0.0012
21	<LOQ	0.086 ± 0.0012	0.006 ± 0.0011

Mean C – concentration for n = 3 (mg/kg) ±SD – standard deviation (mg/kg), LOQ – limit of quantification (mg/kg).

**Figure 1:**
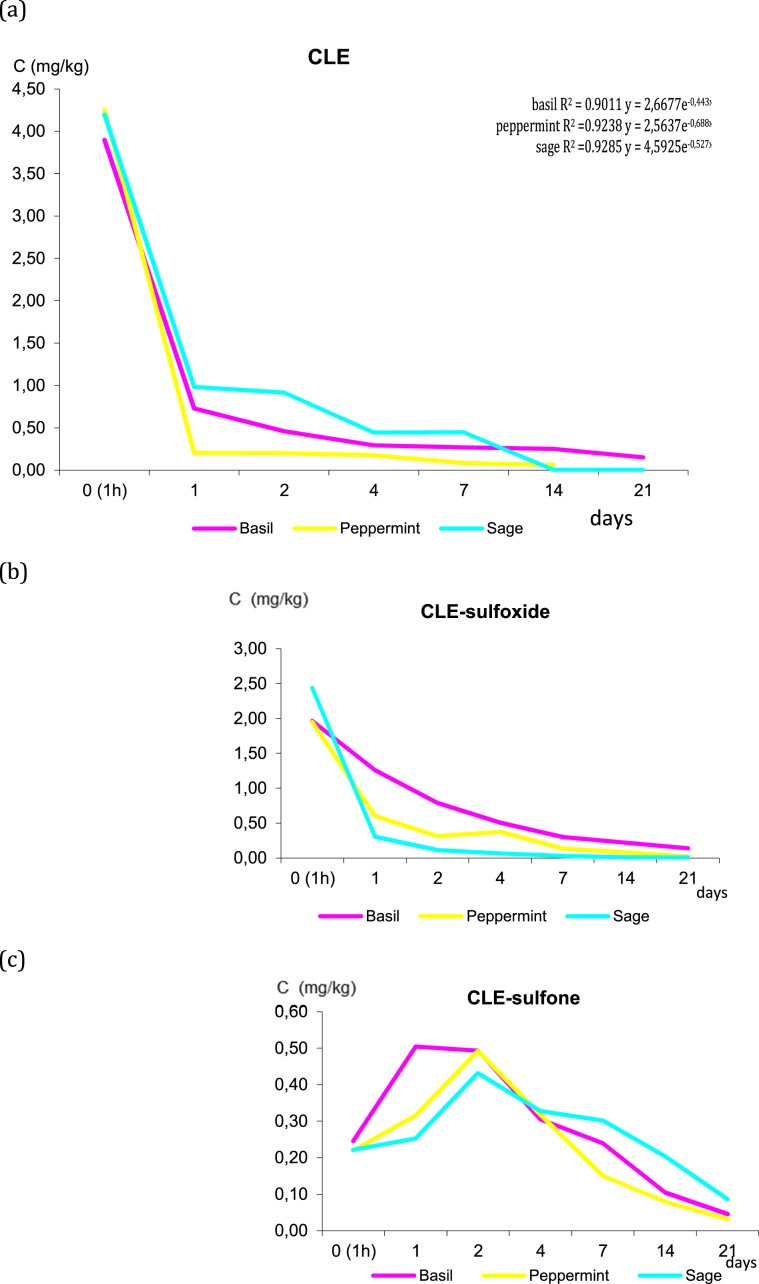
Changes in the concentration of clethodim (CLE) in three species of herbs. (**a**) CLE, (**b**) CLE-sulfoxide, (**c**) CLE-sulfone.

Fate of clethodim and its metabolites in three species of herbal plants is presented in Fig. 1a–c. The residues of total clethodim (CLE, CLE-sulfone and CLE-sulfoxide) in basil, peppermint and sage decreased to 0.336 mg/kg, 0.055 and 0.150 mg/kg, respectively, indicated that were below MRL 0.50 mg/kg established by European Union. Under controlled conditions clethodim rapidly degraded in herbs and converted into its oxidation metabolites CLE-sulfoxide and CLE-sulfon. As presented in the Fig. 1a–c, CLE and CLE-sulfoxide concentration decreased gradually with time elapse giving over 90% to complete degradation, while CLE-sulfone showed a tendency of rapidly increasing first (the highest concentration observed at second day up to 0.50 mg/kg) and continuous decreasing (after 21 days of herbs growth below 0.10 mg/kg). The dissipation rate of CLE in basil, peppermint and sage fitted to first-order kinetics (R^2^ = 0.9011; 0.9238 and 0.9285, respectively) with dissipation equation: y = 2,6677e^−0,443x^ in basil; y = 2,5637e^−0,688x^ in peppermint and y = 4,5925e^−0,527x^ in sage and the half-life of clethodim was 1.56, 1.10 and 1.38 days. Dissipation pathway of CLE in soil samples collected from the herbal plants cultivation proved that CLE rapidly converted to its sulfoxide in all cases (CLE and CLE-sulfone residues lower than LOQ). The initial concentration of CLE-sulfoxide in soil was below 0.250 mg/kg and reduced to below 0.010 mg/kg (more than 95%) after 21 days after application with the half-lives in the range of 4.1–4.5 days.

Our results are consistent with You *et al*.^17^ who showed that clethodim sulfoxide dissipated quickly in rape plant and soil with half-lives of about 4–4.3 days. CLE-sulphone similarly increased initially then decreased in rape plant but was not detected in soil. According to PPDB^33^ clethodim degradation is 3 days in soil (DT50 field). Clethodim sulfoxide and sulphone are mainly formed in soil with estimated maximum occurrence fraction 0.76 and 0.16, respectively, Interestingly, Sandin-Espana *et al*.^34^ reported very rapid photodegradation of clethodim and and its metabolite sethoxydim obtained on silica gel plates in soil and plant surface model systems under simulated solar radiation with half-lives of 1.8 min and 5.0 min, respectively.

### Metabolic profile – fate of spirotetramat

The initial residues of insecticide spirotetramat in basil, peppermint and sage after 1 h of application were 2.619 mg/kg, 3.830 mg/kg and 3.904 mg/kg, respectively (Table 2).

**Table 2:** Fate of spirotetramat in three species of herbs.

Days after treatment	Parent substance	Metabolites			
	Spirotetramat (SPI)	SPI-enol	SPI-ketohydroxy	SPI-monohydroxy	SPI-enol-glucoside
	Mean C ± SD n = 3	Mean C ± SD n = 3	Mean C ± SD n = 3	Mean C ± SD n = 3	Mean C ± SD n = 3
BASIL *Ocimum basilicum* L.					
0 (1 h)	3.619 ± 0.0056	1.952 ± 0.0061	0.162 ± 0.0020	0.008 ± 0.0003	0.007 ± 0.0002
1	1.968 ± 0.0049	1.280 ± 0.0050	0.359 ± 0.0036	0.006 ± 0.0002	0.037 ± 0.0011
2	2.033 ± 0.0051	0.814 ± 0.0048	0.495 ± 0.0065	<LOQ	0.067 ± 0.0022
3	0.447 ± 0.0036	0.542 ± 0.0032	0.416 ± 0.0041	<LOQ	0.063 ± 0.0013
7	0.024 ± 0.0021	0.184 ± 0.0024	0.237 ± 0.0035	<LOQ	0.081 ± 0.0025
14	0.010 ± 0.0012	0.044 ± 0.0021	0.193 ± 0.0023	<LOQ	0.061 ± 0.0039
21	0.002 ± 0.0005	0.027 ± 0.0009	0.095 ± 0.0014	<LOQ	0.072 ± 0.0026
PEPPERMINT *Mentha* × *piperita* L.					
0 (1 h)	3.830 ± 0.0060	2.221 ± 0.0053	0.076 ± 0.0038	0.006 ± 0.0001	0.014 ± 0.0004
1	2.530 ± 0.0051	0.683 ± 0.0024	0.779 ± 0.0051	0.006 ± 0.0002	0.011 ± 0.0002
2	3.330 ± 0.0047	0.238 ± 0.0032	0.666 ± 0.0048	<LOQ	0.005 ± 0.0001
3	0.733 ± 0.0022	0.269 ± 0.0019	0.588 ± 0.0034	<LOQ	0.065 ± 0.0008
7	0.020 ± 0.0008	0.011 ± 0.0002	0.041 ± 0.0025	<LOQ	0.034 ± 0.0003
14	0.008 ± 0.0003	<LOQ	0.009 ± 0.0002	<LOQ	0.037 ± 0.0004
21	0.004 ± 0.0002	<LOQ	0.010 ± 0.0003	<LOQ	0.015 ± 0.0002
SAGE *Salvia officinalis* L.					
0 (1 h)	3.904 ± 0.0069	1.868 ± 0.0033	0.077 ± 0.0008	0.010 ± 0.0003	0.006 ± 0.0002
1	3.001 ± 0.0065	0.699 ± 0.0026	0.538 ± 0.0058	0.007 ± 0.0002	0.005 ± 0.0001
2	2.795 ± 0.0051	0.689 ± 0.0024	0.500 ± 0.0052	<LOQ	0.006 ± 0.0001
3	0.710 ± 0.0013	0.178 ± 0.0016	0.418 ± 0.0021	<LOQ	<LOQ
7	0.134 ± 0.0011	0.161 ± 0.0017	0.091 ± 0.0012	<LOQ	<LOQ
14	0.097 ± 0.0009	0.198 ± 0.0019	0.048 ± 0.0011	<LOQ	<LOQ
21	0.042 ± 0.0006	0.041 ± 0.0008	0.039 ± 0.0009	<LOQ	<LOQ

Mean C – mean concentration for n = 3 (mg/kg) ± SD – standard deviation (mg/kg), LOQ – limit of quantification (mg/kg).

**Figure 2:**
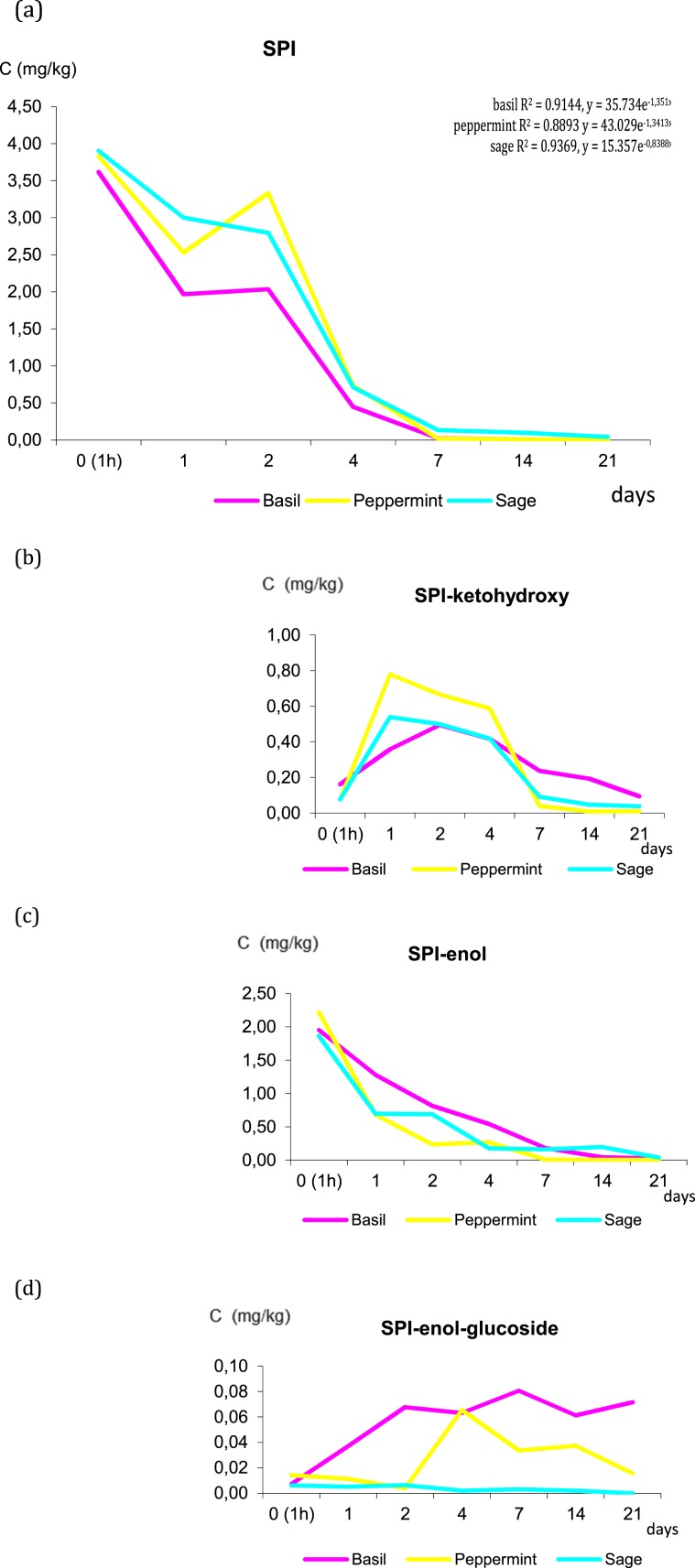
Changes in the concentration of spirotetramat (SPI) in three species of herbs. (**a**) SPI, (**b**) SPI-ketohydroxy, (**c**) SPI-enol, (**d**) SPI-enol-glucoside.

Fate of spirotetramat and its metabolites in herbal plants is presented in Fig. 2a–d. Spirotetramat-monohydroxy was detected in trace amounts (≤0.01 mg/kg) in herbal plants.

The residues of spirotetramat (sum SPI, SPI-enol, SPI-ketohydroxy, SPI-monohydroxy and SPI-enol glucoside) in herbs were lower than EU MRL 4.00 mg/kg. Finally, the concentration of basil, peppermint and sage decreased to 0.196 mg/kg, 0.030 and 0.125 mg/kg, respectively, which indicated that up to 99% of the initial concentrations reduced over the 21 days. Residues dissipated very fast during initial period followed by the first-order equation which provided the best fit to experimental data: y = 35.734e^−1,351x^ basil, y = 43.029e^−1,3413x^ peppermint and y = 15.357e^−0,8388x^ sage and correlation coefficients were R^2^ = 0.9144, 0.8893 and 0.9369, respectively. The half-life t_1/2_ of spirotetramat was 0.51, 0.52 and 0.83 days.

As presented in the Fig. 2b,c, SPI-enol (initial concentration 1. 952 mg/kg in basil, 2.221 mg/kg in peppermint and 1.868 mg/kg in sage) decreased gradually with time, SPI-ketohydroxy (initial concentration 0.162 mg/kg in basil, 0.076 mg/kg in peppermint and 0.077 mg/kg in sage) increased first and then decreased below 0.20 mg/kg in all herbal species. Both, SPI-enol and SPI-ketohydroxy definitively indicated degradation of the parent compound. Meanwhile, SPI-enol-glucoside increased and decreased in varying degrees (concentration range from 0.08 mg/kg to <LOD) and spirotetramat-monohydroxy was detected in trace amounts (≤0.01 mg/kg) in herbal plants. Dissipation study the SPI showed that SPI-ketohydroxy and SPI-enol was only detectable in soil collected from the herbs cultivation with initial deposits of 0.025 mg/kg and 0.061 mg/kg, respectively. The half-lives ranged 2.1–2.8 days and completely reduced (99%) after 21 days from application giving concentrations below 0.005 mg/kg. Residues of SPI-monohydroxy and SPI-enol glucoside metabolites were no detectable in soil (<LOQ).

Similar results reported Zhang *et al*.^35^ showed the first order model for the dissipation of spirotetramat. The half-lives in citrus and soil were in the range of 2.3–8.5 days. However, Łozowicka *et al*.^18^ reported DT50 values of the SPI in *Appiacea* and *Brassicaceae* vegetable roots and leaves were 2.8–2.9 days and 2.1–2.4 days and in soil 0.2 day, respectively.

### Distribution of clethodim and spirotetramat during processing

The change of concentration levels of SPI and CLE were investigated during three processes: washing, drying and washing with drying. The processing factors were assessed for each parent compound, metabolite and also taking into account a sum of parent compound and metabolites expressed as a total residue (Table 3).

**Table 3:** Processing factors of clethodim and spirotetramat and their metabolites in herbs.

Herbal plant			CLE	CLE-sulfone	CLE-sulfoxide	CLE total*	SPI	SPI-ketohydroxy	SPI-enol	SPI-monohydroxy	SPI-enol-glucoside	SPI total*
BASIL *Ocimum basilicum* L.	Initial C (% of parent substance)		3.897	0.246 (6%)	1.965 (50%)	**6.108**	3.619	0.162 (4%)	1.952 (54%)	0.008 (0.2%)	0.007 (0.2%)	**5.748**
	PF	W	0.59	0.84	0.74	**0.49**	0.18	0.68	0.67	0.52	0.54	**0.65**
		D	0.06	2.05	0.30	**0.38**	1.57	0.61	1.69	0.01	0.01	**1.13**
		W + D	0.01	1.63	0.23	**0.14**	0.01	0.39	1.08	—	—	**0.29**
PEPPERMINT *Mentha* × *piperita* L.	Initial C (% of parent substance)		4.251	0.219 (5%)	1.948 (46%)	**6.418**	3.830	0.076 (2%)	2.221 (58%)	0.006 (0.2%)	0.014 (0.4%)	**6.147**
	PF	W	0.69	0.74	0.88	**0.53**	0.45	0.53	0.62	0.05	0.40	**0.68**
		D	0.15	2.12	0.55	**0.36**	1.34	0.45	1.32	—	0.01	**1.50**
		W + D	0.07	1.35	0.34	**0.18**	0.27	0.30	0.13	—	—	**0.27**
SAGE *Salvia officinalis* L.	Initial C (% of parent substance)		4.197	0.221 (5%)	2.437 (58%)	**6.856**	3.904	0.077 (2%)	1.868 (48%)	0.010 (0.2%)	0.006 (0.1%)	**5.865**
	PF	W	0.45	0.90	0.76	**0.54**	0.47	0.85	0.64	0.73	0.43	**0.59**
		D	0.12	2.09	0.54	**0.37**	1.26	0.72	2.60	0.15	0.01	**1.17**
		W + D	0.02	1.36	0.19	**0.15**	0.20	0.31	1.31	—	—	**0.31**

Initial C - concentration obtained after 1 h of pesticide application in mg/kg.

*sum of parent compound and metabolites.

“-” processing factors not estimated due to complete reduction of substance.

PF – processing factor.

W - washing. D - drying. W + D - washing and drying.

### The effect of processing and PFs

The main purpose of the drying operation is to efficiently decrease the high water content of the harvested spices to a safe level in order to get a stable, safe and good quality product^27^.

The processing factor (PF) is the main parameter indicating the changes of pesticide residue level during processing. The calculated PF values for total clethodim after processing were all less than 1 (PFs = 0.14–0.54) indicating reduction in the processed herbs. The initial concentrations of CLE and its two metabolites indicated that 5–6% of clethodim transformed to clethodim sulfone and 46–53% to clethodim sulfoxide. The processing factors for spirotetramat total were calculated for parent compound and its two metabolites B-ketohydroxy and B-enol because B- enol glucoside and B-monohydroxy were not detected in processed samples. The initial concentrations of spirotetramat and its metabolites indicated that 2–4% of spirotetramat transformed to its B-ketohydroxy and 48–58% to B-enol metabolite. PF values of total spirotetramat were in the range of PFs = 0.27–1.50.

Herbs, like other food, undergo the washing step prior to consumption or further processing, it could be expected for pesticides, especially those with limited movement and penetration ability and with a high tendency of loosely maintaining at outer surface, to be removed with reasonable efficiency by this preparatory step^36^. The effectiveness of washing depends on pesticide solubility in water or in different chemical solvents. Thus, clethodim has a good penetrating ability due to its hydrophilic property characterized by high solubility in water (5450 mg/L) and was more easily to remove from basil, peppermint and sage with reduction up to 51% (PFs = 0.49–0.54) than slighty soluble spirotetramat (solubility in water at 20 °C, 55 mg/L) with the loss of residues from 32% to 41% (PFs = 0.59–0.68). The obtained results are consistent with our previous work, in which we have demonstrated that the solubility played a significant role in pesticide removal. Compounds soluble in water (*e.g*. acetamiprid 2950 mg/L PF = 0.43) were more susceptible to elimination during washing fruit and vegetables in contrast to insoluble lambda-cyhalothrin (0.005 mg/L, PF = 0.94)^37^.

Drying is a simple traditional method of herbs preservation^29^. This procedure demonstrated different effectiveness because it has been found to reduce clethodim and clethodim sulfoxide residues and concentrate clethodim sulfone (PFs > 2). However, total clethodim were in the range of PFs = 0.36–0.38. Meanwhile, spirotetramat and spirotetramat B-ketohydroxy residues were concentrated (total PFs = 1.13–1.50). The recorded decreases in pesticides were attributed to evaporation of their residues during drying, while the increase in residue levels was most likely due to weight changes during the process.

This phenomenon may be explained due to temperature in hot air drying caused thermal decomposition of clethodim (degradation point, 100.5 °C). However, spirotetramat PF values of drying were more than 1. Spirotetramat (degradation point, 235 °C) residue content in dried herbs tended to increase because of volatilization of water during processing. This is with agreement with research of Kim *et al*.^38^ who reported that pesticide concentration in processed agricultural product is higher than in raw agricultural product because drying leads to vaporization of water. In our earlier experiment, we have also reported processing factors above one for three pyrethroids (up to PF = 1.76) subjecting berry fruit the high temperatures^37^. Thermal processing did not affect the reduction of alpha-cypermethrin, deltamethrin and lambda-cyhalothrin, compounds with very high degradation point (from 248 °C to 275 °C).

Interestingly, the lowest PFs values in this study were observed when raw commodity was washed before drying. The greatest loss of clethodim and spirotetramat was achieved after drying proceeded by washing, up to 99% (PFs = 0.01–0.07 and PFs = 0.01–0.27, respectively). The residues of clethodim sulfoxide and SPI-enol residues were reduced up to 81% and 70%, respectively. However, clethodim sulfone and SPI-ketoxydroxy after drying regardless of the use of washing in preparation of herbs were more than those in raw plants showing concentration factor above 1. The overall results showed that total clethodim and total spirotetramat residues in dried basil, peppermint and sage were lower than those in unprocessed herbs (up to 86% and 73% of reduction, respectively).

The results demonstrated that washing played very important role in removing residues. This explains that water soluble clethodim (solubility in water at 20 °C, 5450 mg/L and octanol-water partition coefficient logP = 4.14) was more easily removed than spirotetramat (solubility in water at 20 °C, 55 mg/L, logP = 2.51). The larger scope of processing factors proved washing prior to starting the drying process was effective step in removing pesticides. Many factors affected the removal of residue levels e.g. physicochemical properties and systemic character of the pesticides and allowed to make assumptions to explain the difference in the processing factors. These results were similar to the findings of Keikotlhaile^22^ who reported that the concentration of pesticide in processed product is related to solubility, volatility, polarity and thermal decomposition. The loss of residue would mainly attribute to factors of degradation, evaporation and co-distillation^39^. Thus, Lee^40^ reported that hot air drying eliminated 20–30% of azoxystrobin in ginseng products, while Mergnat *et al*.^41^ found that industrial dehydration reduced phosalone levels in apples by over 80%.

Previously, we created a comprehensive database on 160 PFs (processing/pesticide/product combination) for fruit and vegetables after different processing treatments previously. Water (PFs = 0.09–0.94), mechanical (PFs = 0.13–0.32) and thermal (PFs = 0.02–0.57) processing were very effective in reduction of pesticides in berry fruits, Brassica and fruit vegetables^37^. Special attention has been paid to finding the relationship between the efficiency of the technological process and selected physico-chemical properties of active substances. We proved that the key parameters affecting the removal effectiveness were physico-chemical properties of substance i.e. solubility, polarity, degradation point.

The results of the present research focused on herbal matrices might complement European databases of PFs (i.e. EFSA, BfR) which contain data mainly for fruit/vegetables. The overall PFs for CLE-total and SPI-total in the final herb product were in the range of PFs = 0.14–0.18 and PFs = 0.27–0.31, respectively. Significant reduction of the initial concentration levels in basil, peppermint and sage in the range of 69–86% indicates that these herbs are safer to consumers^42^. Thus, combination of washing prior drying is very important practice in herbal plant preservation.

## Conclusion

In the present work, valuable information regarding the metabolic profile of herbicide CLE and insecticide SPI in herbs during plants growing and processing was provided. Our findings predict the safe application of clethodim and spirotetramat at the recommended dosage to protect herbal plants and provide more understanding of herbicide and insecticide behavior in herbs.

These results will supplement the PF database in reference to herbs and might provide more accurate risk assessments of the cyclohexanedione herbicide and the tetramic acid insecticide.

## Material and Methods

### Pesticide standards and solvents

The analytical standards of CLE and its two metabolites CLE sulfone, CLE sulfoxide, and SPI and its four metabolites SPI-enol, SPI-keto-hydroxy, SPI-mono-hydroxy, SPI-enol-glucoside (<99.0% purity) were obtained from Dr. Ehrenstorfer (Augsburg, Germany). All reagents used pesticide residue grade and were obtained from J.T. Baker (Deventer, Holland). More details in Supplementary Information.

### Greenhouse trials

**Figure 3:**
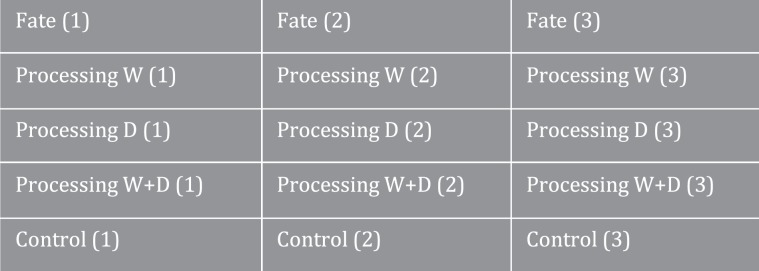
Experimental design of research for each herb. W – washing, D – drying, W + DD – washing plus drying.

The greenhouse trial was performed under controlled conditions located in Bialystok, Poland where no pesticides were applied in the past five years. Herbal plants were cultivated during the agricultural season from June to September 2018. The greenhouse was divided into 15 m^2^ experimental plots, which were separated by a 1 m buffer zone between plots to minimize possible cross-contamination between treatments. The treatment included herbal dynamic test treatment (fate), three terminal residual test treatments (processing) and one control (without pesticides) (Fig. 3). Clethodim (120 kg/m^3^ (13%)) and spirotetramat (100 kg/m^3^ (9.35%)) were applied on basil (*Ocimum basilicum* L.), peppermint (*Mentha* × *piperita* L.) and sage (*Salvia officinalis* L.) were sprayed with manual sprayer at the recommended dose (80 g and 45 g of active ingredient per hectare, respectively) during herb grow at leaf development stage (BBCH 11-29 from the first leaf stage to the end of the tillering phase). The temperature in the greenhouse ranged from 14 to 33 °C and humidity ranged from 75% to 100% from the day of spraying until harvest. The samples from each treatment were collected separately (Fig. 3). A minimum of 200 g of representative herbal plants and soil samples were collected randomly at approximately 1 h (calculated as the initial concentration), 1st, 2nd, 3rd, 7th, 14th and 21th days (terminal concentration) after spraying.

### Sample preparation and processing

**Figure 4:**
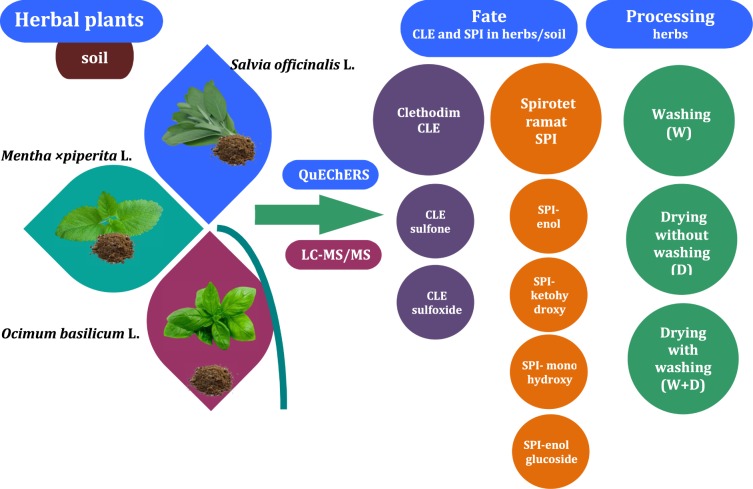
Sample preparation scheme.

The herbal plants immediately after collecting were brought to the analytical laboratory in the same day. The samples for dissipation kinetics without any processing operation were thoroughly homogenized using laboratory blender. The samples for processing study were processed within 24 h after being collected from the greenhouse to determine the changes in clethodim and spirotetramt concentrations in herbs (Fig. 4).

In this study, three methods of decontamination were used: washing (W), drying (D), washing and drying (W + D). For this purpose, samples immediately after sampling were subjected to washing prior drying process, with and without previous pre-treatment (Fig. 1). Herbs were washed under running tap water at temperature app. 20 °C for 1 min, gently shaken to remove excess moisture and then gently dried with a paper towel. Unwashed and washed fresh herbal plants were subjected to the drying processes. Herbs were dried in a oven with ventilation at 50 °C and were proceeded for 5 h. Each treatment was carried out in triplicate. After that, washed (W), dried (D), washed and dried (W + D) samples were blended and taken to analysis.

### Pesticide extraction and purification

The modified quick, easy, cheap, effective, rugged and safe (QuEChERS) method was used for extraction and purification of pesticide residues in fresh/processed herbal and soil samples according to EN 15662:2018^43^. Analytical procedure was previously reported by Rutkowska *et al*.^44^. More details in Supplementary Information.

### Instrumental analysis

Chromatographic separation was carried out with an Eksigent Ultra LC-100 (Eksigent Technologies, Dublin, CA, USA) liquid chromatography (LC-MS/MS) equipped with a Kinetex XB-C_18_ column (1.7 µm, 2.1 × 50 mm) (Phenomenex) analytical column, maintained at 40 °C during the experiments system was operated. More details in Supplementary Information.

### Method validation

Recovery test was performed using spiked herb samples (fresh/dried) at four different concentration levels of selected pesticides (0.001, 0.1, 1.0 and 5.0 mg/kg) in triplicate. Linearity was assessed via the determination coefficient (R^2^) at five points ranging from 0.005 to 2.0 mg/kg. The sensitivity was evaluated by determining the limit of detection (LOD) and the limit of quantification (LOQ) according to Document No. SANTE/1813/2017^34^. More details in Supplementary Information.

### Dissipation kinetics

The dissipation kinetics of CLE and SPI during different species herbal plants growth was estimated using first-order kinetics equation. The persistence of each compound expressed in terms of half-life (t_1/2_) was measured. The following two equations were used: Eq. 1. C_t_ = C_0_e^−kt^ and Eq. 2. t_1/2_ = ln2/k, where C_t_ - concentration of the pesticide residues (mg/kg) at time t (days, C_0_ - initial concentration after 1 h (mg/kg) and k - the dissipation rate constant (per day).

### Processing factor

The processing factor are calculated and considered by the Joint FAO/WHO Meeting on Pesticide Residues (JMPR) as follows: Processing factor (PF = Residue after processing (mg/kg)/Residue before processing (mg/kg). The PF values below 1 (i.e., reduction factor) indicate a reduction of residues in a processed commodity, whereas the values above 1 (i.e., concentration factor) indicate concentration effects from the processing procedures. The unprocessed herb samples obtained from supervised field trial with initial deposits of pesticides were necessary to calculate the processing factors.

## Supplementary information

Supplementary Information.
