# Supplementary material for: Metabolic profile and behavior of clethodim and spirotetramat in herbs during plant growth and processing under controlled conditions
Source: Sci Rep. 2020 Jan 28;10:1323. doi: 10.1038/s41598-020-58130-3 (PMC6987122; doi:10.1038/s41598-020-58130-3)
Supplement: Supplementary file 1 — Supplementary Information. [file 41598_2020_58130_MOESM1_ESM.docx]

**Supplementary Information**

**Metabolic profile and behavior of clethodim and spirotetramat in herbs during plant growth and processing under controlled conditions**

*Magdalena Jankowska*, Piotr Kaczyński, Bożena Łozowicka*

**Table S1.** Characteristics of CLE and SPI and their metabolites [PPDB].

| Parent substance |  | Metabolites |
| --- | --- | --- |
| **Clethodim (CLE)**  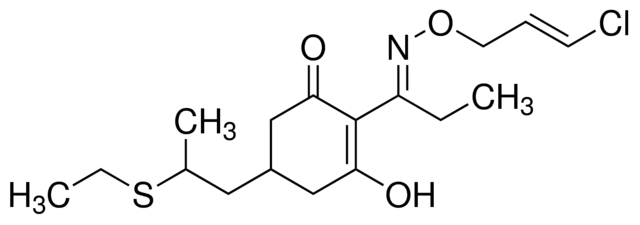  (5*RS*)-2-{(*E*)-1-[(2*E*)-3-chloroallyloxyimino]propyl}-5-[(2*RS*)-2-(ethylthio)propyl]-3-hydroxycyclohex-2-en-1-one  Family of cyclohexanedione herbicides.  Physico-chemical parameters:  Solubility in water at 20°C. 5450 mg/L  Vapor pressure at 20°C. 2.08 * 10^-03^ mPa  Molecular mass 359.92 g/mol  Degradation point. 100.5°C  Melting point -80°C  Boiling point. decomposes before boiling  Octanol-water partition coefficient at pH 7. 20°C logP=4.14  Soil organic carbon absorption coefficient (Koc): 40  Authorised plant protection products in Poland:  Centurion Plus 120 EC  Select Super 120 EC  V-Dim 240 EC  VextaDim 240 EC  MRL = 0.50 mg/kg | CLE sulfone | 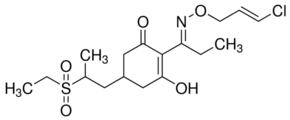 |
|  | CLE sulfoxide | 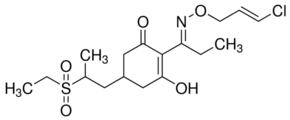 |
| **Spirotetramat (SPI)**  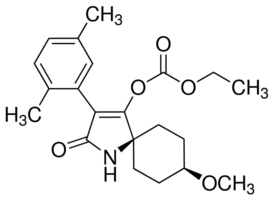  cis-4-(ethoxycarbonyloxy)-8-methoxy-3-(2.5-xylyl)-1-azaspiro[4.5]dec-3-en-2-one  Family of tetramic acid insecticide.  Physico-chemical parameters:  Solubility in water at 20°C. 55 mg/L  Vapor pressure at 20°C. 5.6 * 10^-06^mPa  Molecular mass 373.48 g/mol  Degradation point. 235°C  Melting point. 142°C  Boiling point. decomposes before boiling  Octanol-water partition coefficient at pH 7. 20°C. logP=2.51  Soil organic carbon absorption coefficient (Koc): 289  Authorised plant protection products in Poland:  Movento 100 SC  MRL = 4.00 mg/kg | SPI-enol | 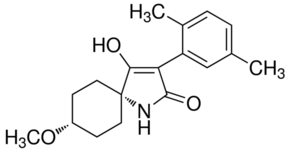 |
|  | SPI-ketohydroxy | 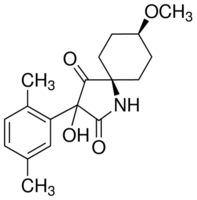 |
|  | SPI-monohydroxy | 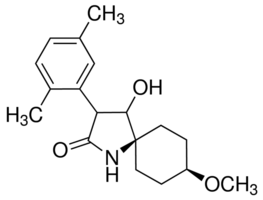 |
|  | SPI-enol-glucoside | 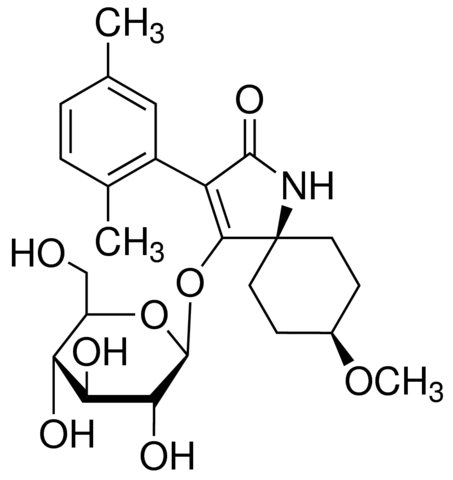 |

**Material and methods**

**Pesticide standards and solvents**

Stock solutions of pesticides and metabolites (around 1000 µg/ml) were prepared in acetonitrile (LC chromatography grade). The standard solutions required for construction of a calibration graph (within the concentration range of 0.005–2.0 µg/ml) were prepared from stock solutions by serial dilution with acetonitrile. Correspondingly, matrix-matched standard solutions were obtained at a series of concentrations by adding blank herb sample extract to each serially diluted standard solution. All solutions were stored in a refrigerator in the dark at 4°C and the working solutions underwent no degradation for 3 months.

**Pesticide extraction and purification**

The modified quick, easy, cheap, effective, rugged and safe (QuEChERS) method was used for extraction and purification of pesticide residues in fresh/processed herbal and soil samples. Briefly, representative 5 g (fresh herb and soil) or 2 g (dried herb) of sample was weighed into a 50 mL PTFE centrifuge tube. In case of dried herb samples,. ten milliliters of cold purified water was added into the tube and shaken by hand for a few seconds to hydrate the samples (standing for 10 min). Then 10 mL of acetonitrile were added to the tube and shaken for 1 min. The mixture was placed on a digital Vortex-Mixer (Velp Scientifica. Usmate. Italy) shaker for 5 min. The tube was placed in a − 20 °C freezer for 15 min. Next, pre-packaged QuEChERS packet of sorbents and salts containing a total of 4 g MgSO_4_, 1 g NaCl, 1 g trisodium citrate dehydrate, and 0.5 g disodium hydrogen citrate sesquihydrate was added and the tube was immediately shaken for 1 min to prevent the formation of crystalline agglomerates during magnesium sulfate hydration and then vortexed at full speed for 1 min. Then the tube was centrifuged at 4500 rpm (Rotina 420R. Hettich) for 10 min.

In case of herb samples the supernatant was transferred to a d-SPE tube containing PSA/ENVI-Carb/MgSO_4_ and then vortexed at full speed for 1 min and centrifuged briefly at 4500 rpm for 10 min. Afterward, 1 mL of the upper layer was filtered through 0.2 mm Nylon syringe filters (15 mm diameter. Agela Technologies. China) into the appropriately labeled autosampler vial for LC-MS/MS analysis.

**Instrumental analysis**

The binary mobile phases were: phase A (water with 0.5 % formic acid and 5 mM ammonium formate) and phase B (methanol with 0.5 % formic acid and 5 mM ammonium formate) at the flow rate 0.5 mL/min. The initial composition of phase A and phase B was 99% and 1% (v/v), respectively and held for 0.5 min. Then linear ramping to 90 % of phase B in 5 min held for 1.5 min and after that returned to the initial composition. The extract volume was 10 µL and chromatographic run time was 12.0 min.

Detection of CLE and SPI and their six metabolites were conducted on a triple-quadrupole mass spectrometry system MS/MS 6500 QTRAP (AB Sciex Instruments. Foster City. CA). equipped with an electrospray ionization source (ESI). The capillary voltage was maintained at 5000 V for positive ion mode and the temperature of the turbo heaters was set at 400°C. As the nebulizer gas (GS1), auxiliary gas (GS2) and curtain gas (CUR) the nitrogen was used at a pressure of 60. 50 and 30 psi, respectively. The nebulizer and collision gas was nitrogen.

Multiple reaction monitoring mode (MRM) was used for pesticides detection. The MRM transitions for the CLE and SPI and their metabolites are given in Table S2.

**Table S2** Parameters for MS/MS of parent substances and their metabolites (MRM transitions).

| Analyte | Retention time (min) | Precursor ion. m/z | Quantification ion transition (CE) | Confirmatory ion transition  (CE) | Declustering potential (V) |
| --- | --- | --- | --- | --- | --- |
| Clethodim (CLE) | 5.90 | 360 | 368 (9) | 164 (10) | 84 |
| CLE-sulfoxide | 5.05 | 376 | 206 (12) | 164 (11) | 91 |
| CLE-sulfone | 5.00 | 392 | 164 (12) | 208 (10) | 96 |
| Spirotetramat (SPI) | 5.40 | 374 | 302 (10) | 330 (15) | 131 |
| SPI-enol | 4.75 | 302 | 216 (10) | 268 (10) | 46 |
| SPI-ketohydroxy | 4.95 | 318 | 300 (10) | 270 (13) | 24 |
| SPI-monohydroxy | 4.45 | 304 | 254 (8) | 272 (12) | 36 |
| SPI-enol-glucoside | 3.55 | 464 | 302 (12) | 270 (14) | 22 |

CE - Collision energy (eV)

**Method validation**

Prior to the extraction step, the spiked samples were allowed to settle for 1 h at room temperature to make sure the target compound penetrate into the matrices evenly. The spiked samples were left without any processing and processed according to the described procedure. Then, the extraction and purification procedure was conducted according to the aforementioned method. The recoveries obtained from the extracted spiked samples were compared with those of the matrix-matched calibration solutions. Calibration curves of the matrix, which were prepared by using aforementioned method, automatically corrected the data for analytical recovery. The relative standard deviation (RSD) was calculated to evaluate precision.

The sensitivity was evaluated by determining the limit of detection (LOD) and the limit of quantification (LOQ) which were estimated by three and ten times the signal-to-noise ratios of the analytes, respectively according to Document No. SANTE/1813/2017.

**References**

PPDB: Pesticide Properties DataBase https://sitem.herts.ac.uk/aeru/ppdb/
